# Platelet Function and Other Indices of Hemostasis in Chronic Liver Disease

**DOI:** 10.4021/gr226e

**Published:** 2010-07-20

**Authors:** Sylvester Chuks Nwokediuko, Obike Godswill Ibegbulam

**Affiliations:** a Department of Medicine, University of Nigeria Teaching Hospital Ituku/Ozalla, Enugu, Nigeria; b Department of Hematology, University of Nigeria Teaching Hospital Ituku/Ozalla, Enugu, Nigeria

**Keywords:** Bleeding time, Hemostasis, Prothrombin time, Partial thromboplastin time, Liver disease

## Abstract

**Background:**

Bleeding time has been used for a long time as a global test of platelet function. Due to a number of pitfalls the test has been losing popularity. This study was designed to determine the prevalence of prolonged bleeding time in Nigerians with chronic liver disease in relation to other indices of hemostasis.

**Methods:**

Bleeding time, platelet count, prothrombin time (PT) and activated partial thromboplastin time (aPTT) were determined in patients with chronic liver disease seen over a twenty-eight-month period. Liver disease severity was graded using Child’s score.

**Results:**

Only 14 of 164 (8.5%) patients with chronic liver disease had prolonged bleeding time while 60 patients (36.6%) had significantly prolonged PT. Thirty seven patients (22.6%) had prolonged aPTT. Bleeding time showed positive correlation with PT and aPTT but negative correlation with platelet count.

**Conclusions:**

Bleeding time is not sensitive in detecting disorders of hemostasis in patients with chronic liver disease although it correlates significantly with other indices of hemostasis.

## Introduction

Platelets are cytoplasmic fragments of the megakaryocyte ranging in size from 2 - 4 microns. The platelet’s contribution to the hemostatic mechanism is through adhesion to the site of injury and aggregation with one another, a process known as primary hemostasis. Secondary hemostasis involves blood coagulation. Studies have demonstrated that abnormalities of platelet function in patients with liver disease arise from intrinsic platelet defects as well as circulating plasma factors [1, 2].

Measurement of skin bleeding time has long been regarded as the global test of platelet function. Despite the shortcomings of bleeding time as a test of platelet function, it is still used in some countries especially the resource- poor countries where more modern tests are not available. Some countries of the world have actually dropped bleeding time as a test of hemostasis [3, 4].

This study was designed to determine the prevalence of abnormal platelet function as measured by bleeding time in patients with chronic liver disease and to correlate the bleeding time in such patients with other indices of hemostasis and liver disease severity.

## Patients and Methods

This was a prospective, cross sectional study of consecutive patients with chronic liver disease who presented to the gastroenterology unit of the University of Nigeria Teaching Hospital (UNTH) Ituku/Ozalla from January 2008 to April 2010. Ethical clearance was obtained from the hospital’s research ethics committee, and informed consent was also obtained from all the participants.

Initial evaluation of the patients included thorough history and detailed physical examination with emphasis on the hepatobiliary system. Ascites was documented and graded as mild, moderate or severe [5, 6]. Hepatic encephalopathy was also documented and graded, using the classification adopted at the 11th World Congresses of Gastroenterology in Vienna 1998 [7]. Patients who took any of the following drugs in the previous week were excluded: aspirin or any nonsteroidal anti-inflammatory drugs, antihistamines, penicillins, cephalosporins, dextran, heparin, beta blockers, sulfonamides and thiazides.

Bleeding time was determined in each patient using the Ivy method [8]. Other laboratory tests performed on the patients included serum bilirubin, liver enzymes (transaminases and alkaline phosphatase), serum protein (total and albumin), prothrombin time (PT), activated partial thromboplastin time (aPTT), and full blood count. The hematological tests were done using an automated hematology analyzer (Sysmex XT 2000i, Sysmex, Japan). The coagulation studies (PT and aPTT) were also done with a coagulation auto-analyzer (Sysmex CA540, Japan). Liver disease severity was assessed using the Child -Turcotte-Pugh scoring system with the parameters of encephalopathy grade, ascites, PT, serum bilirubin and serum albumin [5, 6].

The results were analyzed with the computer software SPSS version 15 and expressed as means and proportions. To compare different indices of hemostasis, correlation coefficient was calculated. P value of less than 0.05 was considered statistically significant.

## Results

There were 164 patients with chronic liver disease consisting of 118 males (72%) and 46 females (28%). Their ages ranged between 23 years and 85 years (mean: 45.91 ± 14.91 years). The mean platelet count of the patients was 231.06×10^9^ ± 152.96×10^9^/L (range: 54 ×10^9^ to 651×10^9^/L). The bleeding time ranged between 2 minutes and 11.2 minutes (mean: 4.21 ± 2.07 minutes). There were 14 patients who had prolonged bleeding time (8.5%). The mean difference between PT of the patients and PT of the control subjects was 2.53 ± 1.83 seconds (range: -0.9 to 8.1 seconds). Sixty patients (36.6%) had PT prolonged by 3 seconds or more. The mean difference between the aPTT of the patients and their controls was 6.57 ± 4.46 seconds (range: 0.9 to 23.2 seconds). The indices of hemostasis are illustrated in Table 1.

**Table 1:** Hemostatic Indices in Patients With Chronic Liver Disease

Index of hemostasis	Mean ± (SD)	Range	Proportion with derangement (%)
Platelet count (x 10^9^)	231.06 (152.96)	54 - 651	42.7
Bleeding Time (minutes)	4.21 (2.07)	2 - 11.2	8.5
PT (seconds prolonged)	2.53 (1.83)	-0.9 - 8.1	36.6
aPTT (seconds prolonged)	6.57 (4.56)	0.9 - 23.2	22.6

Tests of correlation were carried out on the indices of hemostasis (Table 2). For bleeding time and platelet count, the correlation coefficient (γ) was -0.4504, P < 0.0001. Conversely a positive correlation was demonstrated between bleeding time and Child’s score (γ = 0.7111, P < 0.0001). The correlation between bleeding time and PT was positive (γ = 0.7327, P < 0.0001) and the correlation between bleeding time and aPTT was also positive (γ = 0.6403, P < 0.0001). Both PT and aPTT showed a positive correlation with Child’s score (Table 2).

**Table 2:** Correlation Between Indices of Hemostasis in Patients With Chronic Liver Disease

Indices of hemostasis	Pearson (γ)	P value
Bleeding Time vs Platelet count	-0.4504	< 0.0001*
Bleeding Time vs PT	0.7327	< 0.0001*
Bleeding Time vs aPTT	0.6403	< 0.0001*
PT vs Child’s score	0.7913	< 0.0001*
aPTT vs Child’s score	0.8687	< 0.0001*

*=statistically significant; vs=versus

## Discussion

Platelet function as determined by bleeding time in this study did not prove to be a sensitive test for the detection of derangements in hemostasis in patients with chronic liver disease. Only 8.5% of such patients exhibited prolonged bleeding time. However, as expected, the bleeding time correlated positively with other indices of hemostasis such as platelet count, PT and aPTT. Previous studies showed that bleeding time is abnormal in 2.5 to 42% of patients with cirrhosis [9-11].

The explanations for the low prevalence of prolonged bleeding time in chronic liver disease may be derived from the fact that platelet aggregation can undergo either enhancement or inhibition in chronic liver disease depending on a number of factors. Some of these opposing mechanisms may actually operate in the same patient. In humans and in animal models of chronic liver disease, there is a clear hypoaggregability, as demonstrated with in vitro aggregation tests [1, 12, 13]. The molecular mechanisms underlying this intrinsic platelet defect leading to hypoaggregability have been studied extensively. There is evidence supporting a reduced transmembrane signalling in cirrhotic platelets after stimulation with thrombin or collagen [14, 15]. This leads to a decreased activation of phospholipase C, A2 and cyclo-oxygenase / thromboxane synthetase, resulting in decreased thromboxane production [16]. There is also evidence for a decreased arachidonic acid availability for prostaglandin and thromboxane production [17]. Conversely, factors that promote platelet activation include von Willebrand factor (vWF) which is reported to be upregulated in cirrhotic patients [18], and unconjugated bilirubin which is a strong inducer of platelet aggregation [19]. Furthermore, platelet function is known to be affected by the etiology of liver disease, for instance, in cholestatic liver disease, there is some evidence that contrary to other types of cirrhosis, the platelets demonstrate a hyperaggregability [20, 21].

Prothrombin time was deranged in 36.6% of patients with chronic liver disease in this study. When compared to 8.5% which is the prevalence of deranged bleeding time, it becomes obvious that PT is a more sensitive test for detecting hemostatic defects than bleeding time. However, recent findings have questioned the usefulness of PT and aPTT in the determination of risk of bleeding [22-24]. To explain this paradox, it has been argued that the PT and aPTT might be inadequate to reflect the balance of coagulation as it occurs in vivo, especially in chronic liver disease, a condition in which the levels of such naturally occurring anticoagulants as protein C and antithrombin are reduced in parallel with the procoagulants [25].

From the foregoing, it is imperative that simple global tests representing the balance operating in vivo be developed. Thromboelastography is one such technique that can provide continuous observation and tracing of all the hemostatic functions that lead to clot formation and dissolution. It takes into account primary hemostasis, coagulation and fibrinolysis. It is becoming popular in major surgical interventions as liver transplantation and cardiovascular procedures [26, 27]. However, this technique is not yet available in many developing countries where the traditional tests are still in use.

In conclusion, bleeding time as a test of hemostasis has very low yield in detecting abnormalities in patients with chronic liver disease. However, it correlated with platelet number. In view of the shortcomings of the traditional tests of coagulation, simple global tests incorporating the balance between procoagulants and anticoagulants need to be developed.
